# Factors influencing role preferences in decision-making of healthy women with *BRCA1/2* pathogenic variants: subanalysis from a randomised controlled decision coaching trial

**DOI:** 10.1186/s12885-025-13541-1

**Published:** 2025-01-28

**Authors:** Sibylle Kautz-Freimuth, Arim Shukri, Claudia Stracke, Anna Isselhard, Birte Berger-Höger, Anke Steckelberg, Frank Vitinius, Nicola Dikow, Marion Kiechle, Cornelia Meisel, Achim Wöckel, Marion Tina von Mackelenbergh, Rita Schmutzler, Kerstin Rhiem, Stephanie Stock

**Affiliations:** 1 Faculty of Medicine, University of Cologne and Institute for Health Economics and Clinical Epidemiology, University Hospital Cologne, Cologne, Germany; 2 Institute for Health and Nursing Science, Martin Luther University Halle–Wittenberg, Halle (Saale), Germany; 3 Institute for Public Health and Nursing Research, University of Bremen, Bremen, Germany; 4 Faculty of Medicine, University of Cologne and Department of Psychosomatics and Psychotherapy, University Hospital Cologne, Cologne, Germany; 5 Department of Psychosomatic Medicine, Robert Bosch Hospital, Stuttgart, Germany; 6 Institute for Human Genetics, University Hospital Heidelberg, Heidelberg, Germany; 7 Department of Obstetrics and Gynecology, TUM University Hospital, Technical University of Munich, Munich, Germany; 8 Department of Gynecology and Obstetrics, Medical Faculty and University Hospital Carl Gustav Carus, Technische Universität Dresden, Dresden, Germany; 9 National Center for Tumor Diseases/University Cancer Center (NCT/UCC): German Cancer Research Center (DKFZ), Heidelberg, Germany; 10 Faculty of Medicine and University Hospital Carl Gustav Carus, Technische Universität Dresden, Helmholtz-Zentrum Dresden-Rossendorf (HZDR), Dresden, Germany; 11 Department of Obstetrics and Gynecology, University Hospital Würzburg, Würzburg, Germany; 12 Department of Obstetrics and Gynecology, University Hospital Schleswig–Holstein, Kiel, Germany; 13 Faculty of Medicine, University of Cologne and Center for Familial Breast and Ovarian Cancer, University Hospital Cologne, Cologne, Germany

**Keywords:** Activity in decision-making, Actual role in decision-making, *BRCA1/2* pathogenic variants, Control preferences, Decisional conflict, Desired role in decision-making, Hereditary breast and ovarian cancer, Role preferences, Preventive options

## Abstract

**Background:**

Patients who actively engage in their medical decision-making processes can experience better health outcomes. This exploratory study aimed to identify predictors of preferred and actual roles in decision-making in healthy women with *BRCA1/2* pathogenic variants (PVs).

**Methods:**

Women with *BRCA1/2* PVs without a history of breast and/or ovarian cancer were recruited in six centres across Germany. Those returning the baseline questionnaires (T1) were randomly assigned to the intervention or control group (IG, CG). The IG completed a decision-coaching (DC) programme, the CG received standard care. A second survey (T2) followed after 12 weeks. Ordinal regression analyses were performed. Sociodemographic and outcome-related baseline variables were used to identify predictors of (i) desired role at T1 in the total group and (ii) actual role at T2 in the CG and the IG. Role preferences were measured with the Control Preferences Scale.

**Results:**

389 women completed the baseline questionnaires, 191 were randomised to the CG and 198 to the IG. At T1, high decisional conflict (OR 1.016, 95% CI 1.001–1.023, *p* = 0.038) and a negative self-concept (OR 1.030, 95% CI 1.008–1.054, *p* = 0.009) were significant predictors for preferring a more passive role. At T2, high baseline decisional conflict significantly predicted taking a more passive role in the CG, whereas in the IG, baseline decisional conflict showed no influence. Furthermore, in the IG, younger age (OR 1.049, 95% CI 1.001–1.098, *p* = 0.044) and a non-academic education (OR 0.46, 95% CI 0.213–0.775, *p* = 0.006) were identified as significant predictors for taking a more active role.

**Conclusions:**

High initial decisional conflict was identified as an important predictor for preferring and taking a passive role in decision-making among women with *BRCA1/2* PVs. Participating in the DC programme can counteract passivating effects of an initially high decisional conflict and particularly support younger PV carriers and those with lower educational status to take an active role. With this profile, the DC programme expands the existing counselling and care concept to include a measure that can also specifically cover the support needs of younger women and those with a lower education level.

**Trial registration:**

DRKS-ID: DRKS00015527. Registered 30/10/2019.

**Supplementary Information:**

The online version contains supplementary material available at 10.1186/s12885-025-13541-1.

## Background

Active involvement of patients and healthy individuals seeking advice in their medical decisions is associated with positive effects on both the patients’ situation and the doctor-patient relationship. For example, research suggests that patients’ active engagement in their decision-making by taking on an active to collaborative role can promote their autonomy and self-determination, increase their satisfaction and decision-making confidence, and improve their knowledge [1–5]. Further benefits include promoting quality of care, trust in the doctor-patient relationship and adherence to medical treatment [1, 3], which in turn can result in better health outcomes [5, 6]. This emphasises the relevance of the concept of integrating measures into healthcare that support patients in taking on a more active role in their health decision-making processes. As such, these can be valuable and helpful tools on the way to an improved patient-centred healthcare.

Healthy women who are diagnosed with pathogenic variants (PVs) in the genes *BRCA1* and *BRCA*2, are confronted with difficult and far-reaching preventive decisions. Since they are at increased risks of developing breast cancer (BC) with an average lifetime risk of around 70% and ovarian cancer (OC) with average lifetime risks of about 12 to 44% [7, 8], they face the decision whether and if yes, which preventive option to choose and when. Options currently offered in the German context include intensified breast surveillance (IBS), risk-reducing bilateral mastectomy (RRBM) and risk-reducing removal of both ovaries and Fallopian tubes (RRBSO). While IBS identifies BC at early and potentially curable stages in over 80% of cases [9], RRBM significantly decreases BC risk [10, 11] and provides a potential survival benefit for women with *BRCA1* PVs [12]. RRBSO significantly reduces OC morbidity, mortality and overall mortality [13–15]. On the other hand, unfavourable consequences are to consider. As such, IBS does not lower BC rate, and thus, cannot decrease the risk of developing BC. RRBM goes with unwanted effects such as loss of natural breasts and breastfeeding ability, while RRBSO results in loss of natural fertility and potential adverse effects of sudden premature menopause. Weighing pros and cons of each option can be very difficult and depends on individual values and preferences. Decision support tools such as decision aids (DAs) and decision coaching (DC) were developed to assist these women along this challenging decisional journey [16, 17].

Recently, the EDCP-BRCA trial evaluated the effectiveness of a newly developed decision coaching (DC) programme for healthy women with *BRCA1/2* PVs compared with standard care via a randomised controlled trial (RCT) [18]. The DC programme consisted of a nurse-led DC [19] plus an evidence-based DA [20, 21], both especially developed for healthy female *BRCA1/2* PV carriers. One striking result of the EDCP-BRCA trial was that *BRCA1/2* PV carriers who participated in the DC programme were significantly more likely to take an active role in their decision-making process for their preferred preventive strategy compared to women without this intervention [22]. Assuming that *BRCA1/2* PV carriers, who more actively take part in their decision-making processes, will benefit in well-being and health outcomes, it is of clinical relevance to gain deeper insights in whether there are pre-existing factors that might promote or hinder women's desire for an active role and their actual assumption of an active role. Therefore, ordinal regression analyses were conducted in the present study, which is an exploratory subanalysis of the above-mentioned RCT. The first analysis investigated whether baseline variables were associated with the preferred role in decision-making in the total group. The second analysis assessed whether baseline variables were associated with the actual role in decision-making 12 weeks after study inclusion, with separate analyses conducted for the intervention group (IG), which participated in the DC programme, and the control group (CG), which did not. If significant predictors of women's preferred and actual taken roles emerge, this could help to (i) better understand underlying factors promoting or hindering more active decision-making and health behaviours, (ii) further clarify whether specific groups might particularly benefit from the DC programme, and (iii) provide ideas on entry points to better support individuals with an initially more passive attitude.

## Methods

### Study design

The present study used data from the original EDCP-BRCA trial. This RCT was conducted at six centres for familial breast and ovarian cancer in Germany (Cologne, Dresden, Heidelberg, Kiel, Munich, Wurzburg) and assessed the effectiveness of a DC programme for women with *BRCA1/2* PVs compared to standard care (DRKS-ID: DRKS00015527, registered 30/10/2019). A detailed study protocol is published [18]. Ethics approval was obtained from the University Hospital of Cologne (reference number 19–1110).

In the original trial, 413 participants were included in the study. Of these, 389 women returned the baseline questionnaire and were then randomly assigned to the intervention group (IG) or the control group (CG). All participants received standard care. For this, prior to study start, physicians involved in counselling were trained in communication techniques based on the KoMPASS concept [23]. After baseline data collection, the IG additionally participated in the DC programme consisting of a personal nurse-led DC [19] plus an evidence-based DA [20, 21], while the CG received standard care only. Survey points were at baseline (T1) and 12 weeks post study inclusion (T2). The basic content of the DC programme is summarised in Supplementary File 02.

### Study population

We included 389 female *BRCA1/2* PV carriers without a history of BC/OC. These had been included in the original trial and had returned the completed baseline questionnaire. Of these, 191 were randomly assigned to the CG and 198 to the IG. Inclusion criteria were age 25 to 60 years, diagnosed with a definite *BRCA1/2* PV, and sufficient German language skills. Prior to inclusion, all participants had given written informed consent to study participation.

### Baseline independent variables

Baseline variables (T1) were used to identify predictors of (i) the initially preferred role in decision-making and (ii) the role taken 12 weeks post study inclusion. As independent variables, we included parameters that we hypothesised (based on literature) might have an impact on which role women would prefer to take and actually play in their decision making process [24–29] or could be associated with deciding for irreversible risk-reducing surgery [30], which may also indicate a potentially more active attitude towards decision-making [31]. The following socio-demographic and potentially outcome-related independent variables were included: age, educational status (non-academic vs. academic), parity (children yes vs. children no), and baseline data for decision status (not yet decided for preventive option(s) vs. decided), DCS total score, HADS anxiety score, and BRCA self-concept total score. The latter parameters were collected with the following instruments:

- *Decision status* was recorded with the Stage of Decision Making Scale (SDMS) [32]. This 4-item-scale classifies decision-making phases from “not yet thought about the options” to “already made a choice”. For analyses, decision status was dichotomised into (i) “not decided” and (ii) “decided” and the percentages of “undecided” and “decided” women were measured.
- *Decisional conflict* was measured with the Decisional Conflict Scale (DCS) [33, 34] consisting of 16 items, each to be rated from “strongly agree” to “strongly disagree” on a 5-point-Likert-scale. DCS total scores were assessed ranging from 0 (extremely low decisional conflict) to 100 (extremely high decisional conflict).
- *HADS anxiety* is a subscale of the Hospital Anxiety and Depression Scale (HADS) [35, 36] and consists of 7 items, each to be rated on a 4-point-scale from “not at all” to a statement for “strongly present”. Scores range from 0 (extremely mild symptoms) to 21 (extremely severe symptoms).
- *BRCA self-concept* was measured with the BRCA Self-Concept Scale (BRCA-SCS) [37] comprising 17 items, each to be rated on a 7-point-Likert-scale from “strongly agree” to “strongly disagree”. The scale addresses one's own views on personal health, self-esteem, identity and body image from the perspective of carrying a *BRCA1/2* PV. Total scores range from 7 (“very positive self-concept”) to 119 (“very negative self-concept”).

### Outcome variable: control preferences

Outcome variable was the role the women (i) initially preferred at baseline (T1) and (ii) actually took in decision-making 12 weeks post study inclusion (T2). It was measured with the Control Preferences Scale [26, 38, 39]. This 5-point-scale consists of statements that represent preferences for participation in the decision-making processes as “active”, “active-collaborative”, “collaborative”, “passive-collaborative”, and “passive”. Scores range from 1 (“active”) to 5 (“passive”).

For use in the study, the wording of the original CPS items was adapted to the situation of healthy *BRCA1/2* PV carriers who are faced with the decision of which prevention option(s) to choose. In addition, the items of the baseline questionnaire at T1 referred to the preferred, i.e. desired role, whereas the follow-up questionnaire at T2 asked about the role actually taken on. Due to the nature of the survey, the analysis of the actual role played is based on self-reporting by the women and thus reflects the women’s own perception. Supplementary File 03 presents the items as used for evaluation.

### Statistical analyses

Baseline characteristics for the total group, the IG, and the CG were analysed descriptively. Continuous data were depicted by mean and standard deviation, while categorical data were described by frequencies and percentages. Mean differences in continuous variables between IG and CG were tested using the independent two-sided t-test for normally distributed data. Mean differences of scores within groups between follow-ups were assessed applying the dependent two-sided t-test. Nonparametric tests were used for non-normally distributed data. Differences in categorical variables were tested via the chi-square test. No imputation of missing values was conducted.

Ordinal regression analyses were conducted to identify predictors of activity in decision-making. The first analysis was performed on the total group to identify baseline characteristics associated with CPS scores at baseline, representing the desired role in decision-making. The second analysis was conducted separately for the IG and CG to detect baseline characteristics associated with CPS scores at 12 weeks, representing the actual role taken in decision-making. The analysis for the CG identified predictors for decision-making activity without intervention, while the analysis for the IG assessed predictors related to decision-making activity following participation in the DC programme. The subsequent baseline variables were included: age, educational status (non-academic vs. academic), parity (children yes vs. children no), decision status (not yet decided for preventive option(s) vs. decided), DCS total score, HADS anxiety score, and BRCA SCS total score. To assess internal reliability, Cronbach’s alpha was calculated for DCS total score, HADS anxiety score, and BRCA self-concept total score. An α-level of 0.05 was considered significant. All statistical analyses were performed using IBM™ SPSS™ Statistics for Windows, Version 29.0 (IBM Corp, Armonk, NY, USA).

## Results

### Study population

Table 1 summarises baseline characteristics of the study population, collected via the baseline questionnaire at T1. The total group (*n* = 389) showed an average age of 35.3 ± 8.6 years. Strikingly, there was a high proportion of women with an academic degree (46.3%).

**Table 1:** Baseline characteristics of the study population

Characteristic	Total group (*n* = 389)	CG (*n* = 191)	IG (*n* = 198)
Pathogenic variant (PV)^,*^, n (%)	382 (100)	188 (100)	194 (100)
*BRCA1*	190 (49.7)	93 (49.5)	97 (50.0)
*BRCA2*	192 (50.3)	95 (50.5)	97 (50.0)
*Socio-demographic*			
Age, years, mean (SD)	35.3 (8.6)	35.8 (9.2)	34.9 (8.0)
Educational status, n (%)	387 (100)	191 (100)	196 (100)
Non-academic	208 (53.7)	110 (57.6)	98 (50.0)
Academic	179 (46.3)	81 (42.4)	98 (50.0)
Children, n (%)	388 (100)	190 (100)	198 (100)
Yes	194 (50.0)	101 (53.2)	93 (47.0)
No	194 (50.0)	89 (46.8)	105 (53.0)
*Outcome-related*			
CPS: Role in decision-making^a^, n (%)	382 (100)	187 (100)	195 (100)
Active	40 (10.1)	15 (8.0)	25 (12.8)
Active-collaborative	295 (77.2)	144 (77.0)	151 (77.4)
Collaborative	44 (11.5)	26 (13.9)	18 (9.2)
Passive-collaborative	3 (0.8)	2 (1.0)	1 (0.5)
Passive	0 (0.0)	0 (0.0)	0 (0.0)
Decisional status, n (%)	389 (100)	191 (100)	198 (100)
Not decided	239 (61.4)	115 (60.2)	124 (62.6)
Decided	150 (38.6)	76 (39.8)	74 (37.4)
DCS total score, n, mean (SD)	387, 38.2 (19.9)	191, 37.1 (21.0)	196, 39.2 (18.8)
HADS anxiety score, n, mean (SD)	389, 7.1 (3.9)	191, 7.0 (4.0)	198, 7.3 (3.8)
BRCA SCS total score, n, mean (SD)	378, 46.2 (15.6)	186, 45.9 (15.7)	192, 46.5 (15.6)

^a^At T1, the preferred role in decision-making was recorded

*BRCA1/BRCA2* breast cancer gene1/gene2, *BRCA SCS* BRCA self-concept scale, *CPS* control preferences scale, *DCS* decisional conflict scale, *HADS* hospital anxiety and depression scale, *PV* pathogenic variant, *SD* standard deviation

^*^The variant was not documented in seven women

Cronbach’s alpha values were as follows: 0.955 for the DCS total score, 0.833 for the HADS anxiety score, and 0.870 for the BRCA self-concept total score.

### Predictors for the preferred role in decision-making at baseline

At baseline (T1), the total group (*n* = 389) was analysed for factors associated with women's desired roles in decision-making. Table 2 summarises the results. Higher DCS total scores (OR 1.016, [95% CI 1.001; 1.032], *p* = 0.038) and higher BRCA self-concept total scores (OR 1.030, [95% CI 1.008; 1.054], *p* = 0.009) were significant predictors of women preferring a more passive role in decision-making. Conversely, this implies that women experiencing lower decisional conflict and those with a positive self-image were more inclined towards an active role in decision-making. None of the other analysed variables showed significant associations with the preferred role in decision-making.

**Table 2:** Predictors for the preferred role in decision-making at baseline

	**Total Group** (*n* = 389)		
**Outcome variable** **CPS**^**a**^** at T1** (*n* = 382)	**OR**	**95% CI**	***p***
**Independent variables at T1**			
Age	1.029	[0.995; 1.064]	0.100
Educational status: Non-academic^b^	0.961	[0.577;1.599]	0.878
Parity: Having children^c^	0.939	[0.526; 1.677]	0.833
Decision status: Not decided^d^	0.856	[0.476; 1.569]	0.615
Decisional conflict: DCS total score	1.016	**[1.001; 1.032]**	**0.038**
Anxiety: HADS anxiety score	1.013	[0.928; 1.105]	0.722
Self-concept: BRCA SCS score	1.030	**[1.008; 1.054]**	**0.009**

Multiple ordinal regression model with dependent variable CPS at T1. Wald test *p*-values (p), odds ratios (OR) and confidence intervals (CI) are reported for all independent variables included in the analysis

*BRCA SCS* BRCA self-concept scale, *CPS* control preferences scale, *DCS* decisional conflict scale, *HADS* hospital anxiety and depression scale

^a^At T1, the preferred role was recorded

^b^reference: academic

^c^reference: having no children

^d^reference: decided for preventive option

### Predictors for the actual role taken in decision-making at 12 weeks

At 12 weeks post study inclusion (T2), both the CG (*n* = 178) and the IG (*n* = 172) were analysed regarding baseline independent variables associated with women's reported actual roles taken in decision-making. Table 3 summarises the findings.

**Table 3:** Predictors for the actual role taken in decision-making at 12 weeks in CG and IG

	**CG** (*n* = 178)			**IG** (*n* = 172)		
**Outcome variable** **CPS**^**a**^** at T2** (*n* = 350)	**OR**	**95% CI**	***p***	**OR**	**95% CI**	***p***
**Independent variables at T1**						
Age	1.016	[0.977; 1.056]	0.424	1.049	**[1.001; 1.098]**	**0.044**
Educational status: Non-academic^b^	1.612	[0.841; 3.086]	0.150	0.406	**[0.213; 0.775]**	**0.006**
Parity: Having children^c^	0.928	[0.451; 1.910]	0.839	0.783	[0.376; 1.631]	0.514
Decision status: Not decided^d^	1.017	[0.488; 2.118]	0.964	1.514	[0.706; 3.248]	0.287
Decisional conflict: DCS total score	1.031	**[1.013; 1.050]**	**0.001**	1.005	[0.986; 1.026]	0.514
Anxiety: HADS anxiety score	0.997	[0.901; 1.103]	0.952	0.973	[0.863; 1.097]	0.659
Self-concept: BRCA SCS score	1.003	[0.976; 1.030]	0.839	0.987	[0.958; 1.017]	0.391

Multiple ordinal regression model with dependent variable CPS at T2. Wald test *p*-values (p), odds ratios (OR) and confidence intervals (CI) are reported for all independent variables included in the analyses

*BRCA SCS* BRCA self-concept scale, *CG* control group, *CPS* control preferences scale, *DCS* decisional conflict scale, *IG* intervention group, *HADS* hospital anxiety and depression scale

^a^At T2, the role actually taken was recorded

^b^reference: academic

^c^reference: having no children

^d^reference: decided for preventive option

In the CG, a higher baseline DCS total score significantly predicted a more passive role at 12 weeks (OR 1.031, [95% CI 1.013; 1.050], *p* = 0.001). This suggests that among *BRCA1/2* PV carriers who did not participate in the DC programme, initially high decisional conflict was associated with taking a more passive role in decision-making. In contrast to the total group at T1, where higher BRCA self-concept total scores correlated with preferring a more passive role, higher BRCA self-concept total scores after 12 weeks in the CG not receiving the DC programme were not associated with a more passive role assumption.

In contrast to the CG, higher initial DCS total scores in the IG did not predict the role taken at 12 weeks. This means that effects of initially high decisional conflict on role preference, which led to greater passivity in decision-making in the CG, were not observed in the IG following participation in the DC programme.

Furthermore, in the IG, two independent variables predicted roles actually taken. Younger age was significantly associated with taking a more active role in decision-making after 12 weeks (OR 1.049, [95%-CI 1.001; 1.098], *p* = 0.044). Likewise, women with a non-academic status were more likely to take an active role (OR 0.406, [95%-CI 0.213; 0.775], *p* = 0.006). These results indicate that particularly among younger and non-academic women, participation in the DC programme was linked to a more active role taken in their decision-making process.

As younger age was a significant factor for a more active role in decision-making in the IG, it was additionally analysed whether the time periods since genetic testing differed between age groups. There were no statistically significant differences between the groups, indicating that younger and older women had received their genetic diagnoses before comparable time periods. Supplementary File 04 contains the descriptive results.

## Discussion

**Fig. 1:**
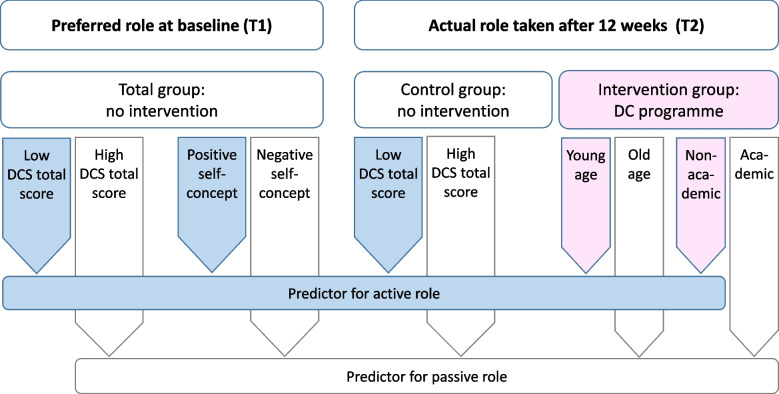
Overview of predictors identified for preferred and actual roles taken in decision-making. Baseline sociodemographic and outcome-related independent variables were used to identify predictors for women’s desired role at baseline (T1) and for the actual role taken 12 weeks post study start (T2). At baseline, high decisional conflict and a negative self-concept predicted a preference for a more passive role in decision-making; vice versa low decisional conflict and positive self-concept predicted preferring a more active role. This suggests that high decisional conflict and negative self-concept are significant predictors for a passive attitude towards one’s own involvement in the decision-making process. After 12 weeks, in the CG, initially high decisional conflict predicted that women would also take a more passive role in decision-making, whereas this association was not present in the IG**.** This suggests that the DC programme contradicts passivating effects of an initially high decisional conflict. In addition, in the IG, young age and non-academic status were independent predictors of taking a more active role in decision-making. This indicates that the DC programme particularly promotes taking a more active role in decision-making among younger women as well as in women with a lower educational status

This study is an exploratory subanalysis of a RCT that assessed the effectiveness of a DC programme in healthy women with *BRCA1/2* PVs. One notable finding of the original trial was that women who participated in the DC programme were significantly more actively engaged in their decision-making process regarding preventive choices compared to women in standard care [22]. In this study, ordinal regression analyses were conducted to identify pre-existing factors or constellations that predict women's preferred and reported actual roles taken in decision-making. Note that it is important to acknowledge the exploratory nature of these analyses and the potential limitations, including the increased risk of Type I errors and demographic biases. Figure 1 provides an overview on which predictors were identified for preferred and actual roles taken in decision-making.

### Preferred roles at baseline

Analysis of baseline characteristics regarding the initially desired role in decision-making showed that with increasing decisional conflict as well as with more negative self-concept, women significantly more desired a passive role in their decision-making process. Vice versa, women with a low decisional conflict and a positive attitude towards their personal situation and self-image were more likely to prefer an active role. To date, research about the association between decisional conflict and role preferences in decision-making with regard to preventive decisions among healthy women with *BRCA1/2* PVs is limited. However, there is indication that in patients with other conditions, such as men with prostate cancer [40] or older people in home care [41], marked decisional conflict was associated with a more passive role in decision-making. With respect to self-concept, there is indication from research that a positive self-concept can support well-being in female *BRCA1/2* PV carriers [42], which could be one reason for being more open to actively engage in decision-making. Nevertheless, to date, little is known about the impact of self-concept on *BRCA1/2* PV carriers' desired role. However, our results are to some extent consistent with findings of a study by Matsen et al. for women diagnosed with BC at young age including women with *BRCA1/2* PVs [28]. The authors identified that a positive view of one's own health significantly correlated with preferring a more active role in decision-making. Further research indicates that signs of a negative self-concept in women with *BRCA1/2* PVs, such as feelings of stigma, can have negative effects by increasing anxiety [43] as well as BC-specific and general distress [44]. These emotional burdens might contribute to one’s level of engagement in decision-making. In fact, anxiety can influence decision-making for risk-reducing surgery, but there are considerable inconsistencies in terms of the effect of low, moderate or high levels of anxiety on promoting, hindering or not influencing the decision [45]. Thus, possible connections between self-concept, emotions and activity in decision-making need to be further explored.

### Actual roles taken at 12 weeks

The investigation of whether baseline characteristics have an influence on the reported actual role taken in decision-making after 12 weeks showed different results for the CG and the IG. Among CG women who had not participated in the DC programme, high decisional conflict at baseline was a significant predictor of taking on a more passive role. Together with the result, that an initially high decisional conflict in the total sample significantly predicted preferring a more passive role, leads to the conclusion that the initial level of decisional conflict considerably influences both desired and actual activity in decision-making, with high decisional conflict lowering actual activity and low decisional conflict increasing actual activity in decision-making.

Remarkably, unlike in the CG in standard care, participating in the DC programme in the IG showed no association between high decisional conflict at baseline and taking a more passive role at 12 weeks. This provides a strong indication that using the DC programme has the potential to counteract passivating effects of an initially high decisional conflict. This, together with another result of the original study, according to which participation in the DC programme significantly reduced decisional conflict [22], indicates that the DC programme positively influences both the decisional conflict itself and its consequences for one's role in decision-making. Recent research found, at least indirectly, a similar coincidence. In a recently updated systematic Cochrane review of 209 RCTs evaluating DAs for their effectiveness, DA use significantly reduced decisional conflict due to feeling uninformed and the proportion of individuals who behaved passive in decision-making [16].

The effect profile found in the present study may be beneficial for clinical practice of counselling and care of women with *BRCA1/2* PVs. For example, by determining the initial level of decisional conflict, counsellors could identify women from the outset who may particularly need and benefit from targeted decision support tools such as the DC programme that can reduce decisional conflict and increase women’s active engagement in decision-making.

Furthermore, in the IG, being at a younger age significantly predicted taking a more active role. This suggests that using the DC programme can support in particular younger women in their active involvement in decision-making. This result also appears to be of clinical relevance considering that younger *BRCA1/2* PV carriers being in their reproductive phase of their lives often face several pressing challenges concerning their health, family, and life planning. For example, in addition to considering risk-reducing surgeries with far-reaching consequences they often feel increased worry and pressure because they need to decide e. g. on partnership, reproductive and breastfeeding wishes, or career planning [31, 46]. Therefore, targeting younger *BRCA1/2* PV carriers in particular would mean that women who tend to experience a higher initial burden and uncertainty might benefit considerably from the DC programme.

While there was no correlation between educational status and the preferred role in the total sample, we identified a clear correlation between the IG’s educational status with the role actually taken. Remarkably, being on a non-academic track was strongly associated with taking on a more active role in decision-making compared to having an academic degree. This suggests that the DC programme specifically supported women with a lower level of education to take a more active role.

Research indicates that both healthy people and cancer patients with a higher level of education show a high intrinsic preference for decision control [47], wish an active role [48], and are intrinsically more involved in decision-making [49]. This indicates they may need less support to participate actively in their medical decision-making processes, while lower educated ones tend to need more support. These indications are confirmed by our result, that being on a non-academic track and then participating in the DC programme predisposed these women to take a more active role in decision-making, whereas this was not the case in women without the intervention. This leads to the conclusion that the DC programme can particularly address a group of women who are less initiative from the outset and may need more support in their decision-making processes. Incorporating the DC programme in the standard care setting, therefore, can considerably broaden the current counselling and care concept for healthy women with *BRCA1/2* PVs. In this way, the DC programme would also meet requirements of the National Action Plan for Health Literacy [50] to facilitate and strengthen patient participation in their health issues. Successful DC implementation can be fostered by commitment of physicians, patients and decision coaches and support from leadership [51].

### Strengths and limitations

One strength of this study is that it is based on a prospective multi-centred RCT including a high number of participants. However, the significance of the results may be limited since the analyses were conducted from an exploratory starting point and the original study focused on the original primary outcome. In addition, the number of independent variables analysed was restricted to those deemed most relevant, thus, possible correlations with other variables were not considered. Furthermore, a bias may arise from the basic profile of the study sample, which is characterised by a relatively young average age and includes nearly double the number of academics compared to the general female population in Germany [52]. Additionally, the exploratory nature of the analyses and the multiple comparisons increase the risk of Type I errors. While some of the odds ratios reported are very slim, even if statistically significant, they should be interpreted with caution. The potential for false positives due to multiple testing warrants a cautious approach when discussing these associations. Future research should aim to validate these findings in more robust, hypothesis-driven studies to confirm their reliability. Nevertheless, the results of the study can serve as a starting point for further prospective research on mechanisms of patient activity in decision-making processes and its improvement. 

## Conclusion

Overall, this study provides a deeper insight in underlying mechanisms that can predispose healthy women with *BRCA1/2* PVs to prefer and take a more active or passive role in their decision-making for their preventive strategy and on how the DC programme specially developed for these women can modulate their active engagement in their decision-making processes. The main results were: (i) an initially high decisional conflict was a significant predictor for preferring and taking a passive role. Having a negative self-concept also was predictive for preferring a passive role, but it was not predictive for the reported actual role taken in the CG who did not participate in the DC programme; (ii) in the IG-women, who participated in the DC programme, younger age and being on a non-academic track were significant predictors for actually taking a more active role in decision-making. Moreover, unlike in the CG, initial decisional conflict had no impact on actual role taken in the IG, indicating that the DC programme can counteract passivating effects of an initially high decisional conflict. Consideration of such predictors could be helpful in clinical practice. Based on our results, one approach could be to identify *BRCA1/2* PV carriers with initially high decisional conflict as well as younger women (with potentially more unresolved issues) and women with non-academic education and to provide them with more targeted support from the outset, e. g. through psychological counselling and programmes to support their decision-making and active engagement, such as the present DC programme. Thus, specifically *BRCA1/2* PV carriers with burdening factors that foster passive attitudes towards decision-making could particularly benefit from the DC programme. However, further research is required to substantiate these results in prospective studies.

## Supplementary Information

Supplementary Material 1.

Supplementary Material 2.

Supplementary Material 3.

Supplementary Material 4.

## Data Availability

The datasets generated and/or analysed during the current study are not publicly available and cannot be provided to third parties due to the following reasons. Although the participants of the original EDCP-BRCA study have given their consent to the analysis and publication of the study results, unfortunately their consent to the transfer of data to third parties was not obtained. It is, therefore, not possible for us to pass on anonymised data to interested third parties.

## Abbreviations

BC: Breast cancer
*BRCA1/2*: Breast cancer gene 1/gene 2
BRCA SCS: BRCA self-concept scale
CG: Control group
CI: Confidence interval
CPS: Control preferences scale
DA: Decision aid
DC: Decision coaching
DCS: Decisional conflict scale
DRKS: Deutsches Register Klinischer Studien (German Register of Clinical Trials)
EDCP-BRCA: Evaluation of a decision coaching programme for structured decision support for preference-sensitive decisions in the context of risk-adapted prevention in *BRCA1/2* mutation carriers
G-BA: Gemeinsamer Bundesausschuss (Federal Joint Committee)
HADS: Hospital anxiety and depression scale
IBS: Intensified breast surveillance
IG: Intervention group
n: Number
OC: Ovarian cancer
OR: Odds ratio
PV: Pathogenic variant
RCT: Randomised controlled trial
RRBM: Risk-reducing bilateral mastectomy
RRBSO: Risk-reducing bilateral salpingo-oophorectomy
SD: Standard deviation
SDMS: Stage of decision making scale
