# Supplementary material for: Factors influencing role preferences in decision-making of healthy women with BRCA1/2 pathogenic variants: subanalysis from a randomised controlled decision coaching trial
Source: BMC Cancer. 2025 Jan 28;25:164. doi: 10.1186/s12885-025-13541-1 (PMC11776258; doi:10.1186/s12885-025-13541-1)
Supplement: Supplementary file 1 — Supplementary Material 1. [file 12885_2025_13541_MOESM1_ESM.docx]

**Supplementary File 01**

**Questions (Q) and response options in the T1 and T2 questionnaires
on sociodemographic data, type of pathogenic variants and the instruments used in this study**

*(English translation)*

**Socio-demographic and medical baseline variables**

Q: In this section, we ask you to provide some information about yourself. Please answer each question with the appropriate statement or check the appropriate box.

1. How old are you?
   _______ years
2. Do you have children?
   a. Yes
   b. No

3. What is your highest successfully completed level of education?

- 1. No qualifications
  2. General secondary school certificate
  3. Intermediate secondary school certificate
  4. University entrance certificate
  5. University degree

4. In which gene have you been diagnosed with a mutation?
*(Multiple answers possible)*

- 1. *BRCA1*
  2. *BRCA2*
  3. Other _________

**Decision status: Stage of Decision Making Scale (SDM-S)**

Q: During the genetic test and risk consultation, it was discussed with you that there are different preventive options to deal with an increased risk of breast and ovarian cancer. These include intensified breast surveillance and risk-reducing surgeries. In this section, we ask you to indicate which stage of the decision-making process for one of the possible preventive measures you are currently in. Please read the following statements and tick the option that best applies to you. Please select only one option.

1. I have not yet thought about the options.
2. I am considering the options.
3. I am close to choosing one option.
4. I have already made a choice.

*Reference SDM-S: O’Connor A, Grant F. User Manual - Stage of Decision Making Scale. Ottawa Hospital Research Institute, Ottawa, Canada 2000, updated 2003. //decisionaid.ohri.ca/docs/develop/User_Manuals/UM_Stage_Decision_Making.pdf*.

**Decisional Conflict: Decisional Conflict Scale (DCS)**

Q: In this section, we would like to learn about the attitudes and feelings you have as a carrier of a *BRCA1/2* mutation towards your preventive options. Please read the following statements carefully. Then rate the extent to which the statements apply to your current decision-making situation (from “strongly disagree” to “strongly agree”)

Response options: Strongly agree (0) – agree (1) – neither agree nor disagree (2) – disagree (3) – strongly disagree (4)

1. I know which options are available to me.
2. I know the benefit of each option.
3. I know the risks and side effects of each option.
4. I am clear about which benefits matter most to me.
5. I am clear about which risks and side effects matter most to me.
6. I am clear about which is most important to me (the benefits or the risks and side effects).
7. I have enough support from others to make a choice.
8. I am choosing without pressure from others.
9. I have enough advice to make a choice.
10. I am clear about the best choice for me.
11. I feel sure about what to choose.
12. This decision is easy for me to make.
13. I feel I have made an informed choice.
14. My decision shows what is important to me.
15. I expect to stick with my decision.
16. I am satisfied with my decision.

*Reference DCS: O’Connor A. User Manual - Decisional Conflict Scale. Ottawa Hospital Research Institute, Ottawa, Canada 1993, updated 2010. //decisionaid.ohri.ca/docs/develop/User_Manuals/UM_Decisional_Conflict.pdf.*

**Preferred and actual role: Control Preferences Scale (CPS)**

***In the T1 questionnaire*** ***(preferred role)***

Q: The following section deals with the role that you, as the person seeking advice, would like to play in the decision-making process in favor of a preventive measure. Below, you will see five different ways in which people seeking advice would like to be involved in this decision. Please read the corresponding statement for each illustration and tick the option that best reflects how you would like to make your decision. Please tick only one of the five options.

1. I would like to decide for myself which preventive measure I will receive.
2. I would ultimately like to decide on my preventive measure after I have seriously considered my doctor’s opinion.
3. I would like my doctor and I to share responsibility for deciding which preventive measure is best for me.
4. I would like my doctor to make the final decision about my preventive measure, but to take my opinion into account.
5. I would like to leave all decisions regarding my preventive measure to my doctor.

***In the T2 questionnaire*** ***(role actually taken)***

Q: The following section deals with the role that you, as the person seeking advice, have played in the decision-making process for a preventive measure. Below you will see five different ways in which people seeking advice contribute to this decision. Please read the corresponding statement for each illustration and tick the option that best reflects how you made your decision. Please tick only one of the five options.

Afterwards, please indicate how satisfied you were with your role in the decision-making process.

1. I have decided for myself which preventive measure I receive.
2. I have decided on my preventive measure after seriously considering my doctor’s opinion.
3. My doctor and I shared the responsibility for deciding which preventive measure is best for me.
4. My doctor made the final decision about which preventive measure to choose, but seriously considered my opinion.
5. I left all decisions regarding my preventive measure to my doctor.

Additional question (*not part of the CPS*):

1. I was satisfied with my role taken in the decision-making process.
   1. Strongly diasagree
   2. Disagree
   3. Neither agree nor disagree
   4. Agree
   5. Strongly agree

*Reference CPS: Degner LF, Sloan JA, Venkatesh P. The Control Preferences Scale. Can J Nurs Res Rev Can Rech En Sci Infirm. 1997;29:21–43.
Note: The wording of the items was adapted to reflect the preferred and actual roles associated with the decision-making process for preventive options for BRCA1/2 PV carriers.*

**Anxiety symptoms: Hospital Anxiety and Depression Scale, HADS-Anxiety**

Q: The following section is about your general and emotional well-being. For each statement, please tick the answer that best applies to yourself in the last week. Please tick only one box for each statement and please do not skip any statements. Please do not think long and choose the answer that seems most relevant to you straight away.

*Note: As the HADS is not free of use, we list the seven categories that are included in the HADS anxiety subscale and that were asked about in the questionnaire:*

1. Nervous tension
2. Concerns and worries
3. Concerns and worries
4. Motor tension; relaxation deficits
5. Motor tension; relaxation deficits
6. Motor tension; relaxation deficits
7. Panic attacks

*References HADS: Zigmond AS, Snaith RP. The Hospital Anxiety and Depression Scale. Acta Psychiatr Scand. 1983;67:361–70. Hermann-Lingen C, Buss U, Snaith RP, Zigmond AS (2011). Hospital Anxiety and depression Scale - German Version (HADS-D). Manual. Hans-Huber, Hogrefe AG, Bern.*

**Self-concept: BRCA-self concept scale (BRCA SCS)**

Q: In the following section, you will find a list of statements that some people make about themselves. Please read the statements carefully. Then rate how much you agree with the statements (from 1 “Strongly disagree” to 7 “Strongly agree”).

Response options: Strongly disagree (1) – (2) – (3) – (4) – (5) – (6) – (7) strongly agree

1. I am hopeful about myself in the future.

2. I am able to deal with my test result.

3. I feel my body has betrayed me.

4. (Since I know the genetic test result,) I feel like a walking time bomb.*

5. I feel different from others my age.

6. I know my body well.

7. I feel guilty that I might pass on an elevated cancer risk to my children.

8. I feel isolated because of my test result.

9. I feel I have lost my sense of privacy (due the test result).*

10. I think about my test result a lot.

11. I am worried that cancer will be found when I go for screening.

12. I feel labelled.

13. I feel burdened with this information (my test result).*

14. I distrust my body.

15. I am in control of my health.

16. My test result gets in the way of who I really am.

17. I have become more secretive.

*Reference BRCA self-concept:*

*Esplen MJ, Stuckless N, Hunter J, Liede A, Metcalfe K, Glendon G, et al. The BRCA Self-Concept Scale: a new instrument to measure self-concept in BRCA1/2 mutation carriers. Psychooncology. 2009;18:1216–29.*

*Note: *In some items, the reference to the test result was added to the original wording.*
