# Supplementary material for: Factors influencing role preferences in decision-making of healthy women with BRCA1/2 pathogenic variants: subanalysis from a randomised controlled decision coaching trial
Source: BMC Cancer. 2025 Jan 28;25:164. doi: 10.1186/s12885-025-13541-1 (PMC11776258; doi:10.1186/s12885-025-13541-1)
Supplement: Supplementary file 2 — Supplementary Material 2. [file 12885_2025_13541_MOESM2_ESM.docx]

**Supplementary File 02**

**Information on the content of the DC programme**

The decision-coaching (DC) programme evaluated in the original EDCP-BRCA trial [1] as well as in the present subanalysis consists of a nurse-led DC [2] plus an evidence-based decision aid (DA) [3, 4], both especially developed for healthy women with *BRCA1/2* PVs. The aim is to support these women in their decision-making process when choosing the most suitable preventive option and the most appropriate time for it.

The evidence-based decision aid (DA) is a non-directive tool that serves to support medical consultation and risk counselling. It was developed in a structured process based on the IPDAS criteria [3]. This involved several review phases by women with *BRCA1/2* PVs who were not involved in the creation of the DA, as well as by medical, psychological and self-help experts. The DA provides layperson-friendly and evidence-based information about *BRCA1/2* PVs and the associated risks for breast and ovarian cancer, the prevention options available in the German healthcare system, their benefits and risks, and the associated probabilities. The DA is designed to help healthy *BRCA1/2* PV carriers understand their risk profile and the benefits and risks of each option, to realistically assess the importance of the options for themselves and gain more clarity about their wishes, values and preferences. As part of the DC programme, the DA is provided to the women approximately one to two weeks before they take part in the nurse-led DC.

The nurse-led DC is a non-directive personal exchange between the woman and a health professional (mostly a nurse) qualified by training. The aims are to address open questions, to clarify further information and support needs, to clarify values and preferences with regard to the individual options for action, to support the woman in making an informed decision and prepare the woman concerned for the implementation of her decision. In detail, this means that those seeking advice are prepared to play an active role in a doctor’s consultation in terms of participatory decision-making. During decision coaching, women are also given the opportunity to ask questions and to obtain further information [5, 6].

In the framework of the DC programme used, a nurse qualified in DC elicits open questions and further information and support needs and instructs the woman seeking advice in one to two sessions on how to carry out the following steps, based on the Ottawa Decision Support Framework [7, 8]:

- Clarification of the decision-making situation
  - What decision(s) are involved?
  - Where is the woman in her decision-making process?
- Recording the need for support
  - Exploration of the woman’s knowledge regarding the risks posed by the *BRCA1/2* PV

and her preventive options

- Information and counselling about the individual risks and options for action
  - What alternatives can the woman choose from?
- Support in clarifying individual values and preferences
  - Which factors are important for the woman in her decision-making process?
- Weighing up the alternatives and formulating the decision preference
- Clarification of the feasibility of the alternative courses of action in the woman’s life situation
- Support in communicating the decision during the doctor’s consultation

The medical content in the DA and further material used by the decision coaches (nurses) is based on evidence-based information provided by the currently available S3 and S2 guidelines for the German health care context and on further research data with a high level of evidence.

Contents addressed include:

- Risk profile for breast and ovarian cancer, as outlined in the medical consultation, including lifetime risks, age-related risks, individual risk profile (taking into account the result of a genetic test, other genetic aspects (e.g. polygenic risk score PRS) and other non-genetic risk factors)
- Preventive options: intensified breast surveillance, risk-reducing bilateral mastectomy, risk-reducing bilateral salpingo-oophorectomy
- Benefits and harms of the options, importance for the individual, and personal wishes, values and preferences in terms of each option
- Possible other aspects that may be connected to woman's decision-making for her preventive strategy, such as current life situation and planning

**References**

1. Stock S, Isselhard A, Shukri A, Kautz-Freimuth S, Redaèlli M, Berger-Höger B, et al. Decision coaching for healthy women with BRCA1/2 pathogenic variants. Deutsches Ärzteblatt. 2024. https://www.aerzteblatt.de/int/archive/article?id=239795. Accessed 25 Jun 2024.

2. Berger-Höger B, Vitinius F, Fischer H, Beifus K, Köberlein-Neu J, Isselhard A, et al. Nurse-led decision coaching by specialized nurses for healthy BRCA1/2 gene mutation carriers - adaptation and pilot testing of a curriculum for nurses: a qualitative study. BMC Nurs. 2022;21:42.

3. Kautz-Freimuth S, Redaèlli M, Rhiem K, Vodermaier A, Krassuski L, Nicolai K, et al. Development of decision aids for female BRCA1 and BRCA2 mutation carriers in Germany to support preference-sensitive decision-making. BMC Med Inform Decis Mak. 2021;21:180.

4. Kautz-Freimuth S, Redaèlli M, Shukri A, Kentenich H, Simic D, Mildenberger V, et al. Effectiveness of evidence-based decision aids for women with pathogenic BRCA1 or BRCA2 variants in the german health care context: results from a randomized controlled trial. BMC Med Inf Decis Mak. 2023;23:223.

5. Stacey D, Murray MA, Légaré F, Sandy D, Menard P, O’Connor A. Decision coaching to support shared decision making: a framework, evidence, and implications for nursing practice, education, and policy. Worldviews Evid Based Nurs. 2008;5:25–35.

6. Jull J, Köpke S, Smith M, Carley M, Finderup J, Rahn AC, et al. Decision coaching for people making healthcare decisions. Cochrane Database Syst Rev. 2021;2021.

7. Légaré F, O’Connor AC, Graham I, Saucier D, Côté L, Cauchon M, et al. Supporting patients facing difficult health care decisions: use of the Ottawa Decision Support Framework. Can Fam Physician Med Fam Can. 2006;52:476–7.

8. Stacey D, Légaré F, Boland L, Lewis KB, Loiselle M-C, Hoefel L, et al. 20th Anniversary Ottawa Decision Support Framework: Part 3 Overview of Systematic Reviews and Updated Framework. Medical decision making. 2020;40:379–98. doi:10.1177/0272989X20911870.
