# Supplementary material for: Factors influencing role preferences in decision-making of healthy women with BRCA1/2 pathogenic variants: subanalysis from a randomised controlled decision coaching trial
Source: BMC Cancer. 2025 Jan 28;25:164. doi: 10.1186/s12885-025-13541-1 (PMC11776258; doi:10.1186/s12885-025-13541-1)
Supplement: Supplementary file 3 — Supplementary Material 3. [file 12885_2025_13541_MOESM3_ESM.docx]

**Supplemental File 03**

**Description of the Control Preferences Scale (CPS), as used in the EDCP-BRCA trial**

The control preference construct is defined as "the degree of control an individual wants to assume when decisions are being made about medical treatment." Selectable preferred roles range from the individual making the decisions (active role), through the individual making the decisions jointly with the physician (active-collaborative role; collaborative role; passive-collaborative role), to the physician making the decisions (passive role). The CPS has proven to be a clinically relevant, easily administered, valid, and reliable measure of preferred roles in healthcare decision making. The scale is a validated tool to gauge individuals' preferences for involvement in medical decision-making. The scale consists of five statements representing various levels of control in healthcare choices, it has often been used in studies on decision support and shared decision-making.

In the EDCP-BRCA trial [1], the items’ wording of the original CPS [2] was adapted to the decision-making situation of healthy *BRCA1/2* PV carriers who face the decision which preventive option(s) to choose. The CPS for the T2 survey was also modified to ask the women about their actual role taken at T2. The adapted CPS was then used to assess the women’s

1. preferred (desired) role in the decision-making process at study start (=baseline; T1)
2. actual role taken in the decision-making process 12 weeks post study start (=follow-up; T2).
   Since the CPS at T2 asks about the self-reported role actually assumed, the statements on this are about the actual role taken as the women perceived for themselves.

In the study’s baseline questionnaire at T1, respondents indicated their preferred role in the decision-making process from the following options:

1. I would like to decide for myself which preventive measure I will receive. (active role)
2. I would ultimately like to decide on my preventive measure after I have seriously considered my doctor’s opinion. (active-collaborative role)
3. I would like my doctor and I to share responsibility for deciding which preventive measure is best for me. (collaborative role)
4. I would like my doctor to make the final decision about my preventive measure, but to take my opinion into account. (passive-collaborative role)
5. I would like to leave all decisions regarding my preventive measure to my doctor. (passive role)

In the study’s follow-up questionnaire at T2, respondents indicated their actually taken role in the decision-making process as they had perceived it personally from the following options:

1. I have decided for myself which preventive measure I receive. (active role)
2. I have decided on my preventive measure after seriously considering my doctor’s opinion. (active-collaborative role)
3. My doctor and I shared the responsibility for deciding which preventive measure is best for me. (collaborative role)
4. My doctor made the final decision about which preventive measure to choose, but seriously considered my opinion. (passive-collaborative role)
5. I left all decisions regarding my preventive measure to my doctor. (passive role)

**References**

[1] Stock S, Isselhard A, Shukri A, Kautz-Freimuth S, Redaèlli M, Berger-Höger B, et al. Decision coaching for healthy women with BRCA1/2 pathogenic variants. Dtsch Ärztebl 2024. https://www.aerzteblatt.de/int/archive/article?id=239795 (accessed June 25, 2024).

[2] Degner LF, Sloan JA, Venkatesh P. The Control Preferences Scale. Can J Nurs Res Rev Can Rech En Sci Infirm 1997;29:21–43.
