# Supplementary material for: Factors influencing role preferences in decision-making of healthy women with BRCA1/2 pathogenic variants: subanalysis from a randomised controlled decision coaching trial
Source: BMC Cancer. 2025 Jan 28;25:164. doi: 10.1186/s12885-025-13541-1 (PMC11776258; doi:10.1186/s12885-025-13541-1)
Supplement: Supplementary file 4 — Supplementary Material 4. [file 12885_2025_13541_MOESM4_ESM.docx]

**Supplementary File 04**

**Table S1: Date and time since genetic testing in different age groups**

| **Date of  genetic testing** | ***Approximate time since genetic testing*** | **A: age  ≤35 years** | | **B: age  36 - 45 years** | | **C: age  ≥46 years** | | ***p* value** |
| --- | --- | --- | --- | --- | --- | --- | --- | --- |
|  |  | n | (%) | n | (%) | n | (%) |  |
| 01/01/2005 - 31/12/2009 | *ca. 16 - 12 years* | 0 | 0 | 5 | 4,6 | 1 | 1.9 | .383 (B vs C) |
| 01/01/2010 - 31/12/2014 | *ca. 11 - 7 years* | 21 | 9.3 | 11 | 10.1 | 4 | 7.4 | .816 (A vs C) .816 (B vs A)  .816 (B vs C) |
| 01/01/2015 - 31/12/2019 | *ca. 6 - 2 years* | 89 | 39.4 | 42 | 38.5 | 17 | 31.5 | .881 (A vs B)  .567 (A vs C)  .567 (B vs C) |
| from 01/01/2020 | *<1 - <2 years* | 116 | 51.3 | 51 | 46.8 | 32 | 59.3 | .436 (A vs B) .436 (C vs A)  .402 (C vs B) |
| Total |  | 226 | 100 | 109 | 100 | 54 | 100 |  |
| *Note: The first participant was recruited on 18/11/2019; the last participant was recruited on 27/10/2021.  p values were calculated by the z-test for proportions; vs: versus* | | | | | | | | |

In order to investigate whether there are differences in the time periods since the genetic tests were carried out in women of different ages, the time periods since genetic testing were analysed in the age groups ≤35 years, 36-45 years and ≥46 years.

Table S1 shows the proportions of women who received their genetic test result in the three age groups different lengths of time ago. There were no statistically significant differences between the three age groups in terms of the length of time since the genetic testing.

Most of the women in each age group received their genetic test result in the period from less than around one year to less than around two years before study inclusion. This was the case for 51.3% of the ≤35 age group, for 46.7% of 36- to 45-year-olds, and for 59.3% of the ≥46 age group, respectively.
